# High Serum Alkaline Phosphatase Flare after First-Line Androgen Deprivation Therapy Predicts Poor Prognosis in Metastatic Prostate Cancer Patients Treated with Second-Generation Androgen Receptor Targeted Therapy

**DOI:** 10.1155/2021/5574067

**Published:** 2021-04-08

**Authors:** Satoko Kojima, Hiroshi Masuda, Takahito Suyama, Kyokushin Hou, Kousuke Mikami, Kazuhiro Araki, Yukio Naya

**Affiliations:** Department of Urology, Teikyo University Chiba Medical Center, Ichihara, Japan

## Abstract

**Objectives:**

To determine whether an alkaline phosphatase (ALP) flare after androgen deprivation therapy (ADT) is associated with the treatment response in castration-resistant prostate cancer (CRPC) and predicts the prognosis of metastatic prostate cancer (PCa) patients.

**Methods:**

One hundred and nineteen patients diagnosed with metastatic PCa between 2008 and 2017 were retrospectively studied. The ALP flare ratio was calculated as the ratio of ALP levels 1 month after beginning ADT to ALP levels at diagnosis. The association of the ALP flare ratio with the prostate-specific antigen (PSA) response to CRPC treatment (second-generation androgen receptor targeted therapy (ART) or docetaxel), time to CRPC, and overall survival (OS) were investigated.

**Results:**

The time to CRPC and OS was significantly longer in patients with an ALP flare ratio less than 1.33 compared to a ratio more than 1.33. No difference in PSA response was seen regarding the ALP flare ratio in both ART and docetaxel treatment. Second-generation ART-treated patients with a low ALP flare ratio showed longer OS than those with a higher ALP flare ratio (*p*=0.0367). However, no difference was seen between a high and low ALP flare ratio (*p*=0.8054) in docetaxel-treated patients. The ALP flare ratio was the most significant prognostic factor for OS (*p* < 0.0001).

**Conclusions:**

A higher ALP flare ratio after first-line ADT was a significant prognostic factor in metastatic PCa, especially in patients treated with second-generation ART for CRPC. Chemotherapy for patients with a higher ALP flare ratio 1 month after induction of ADT may be a clinically relevant decision.

## 1. Introduction

Prostate cancer (PCa) is the second leading cause of cancer death in men in Western countries [1]. Widely used prostate-specific antigen (PSA) screening has decreased the number of *de novo* metastatic PCa cases at diagnosis, but some metastatic PCa patients are still identified [2]. Androgen deprivation therapy (ADT) is an effective treatment for most PCa patients; however, these patients eventually progress to castration-resistant prostate cancer (CRPC). The median overall survival (OS) of metastatic PCa patients is approximately 3–6 years, depending on the tumor volume, visceral metastasis, and other prognostic factors [2, 3]. Docetaxel and second-generation androgen receptor targeted therapy (ART) are effective treatments not only for CRPC but also for metastatic hormone-sensitive PCa (HSPC) [4–7]. Which patients would benefit from chemotherapy as initial treatment is not clear. To predict the response or prognosis of HSPC, a reliable predictive marker is needed. A time to CRPC less than 12 months and a PSA nadir value (<0.2 ng/mL) at 6 months after induction of ADT are well-known prognostic predictors [3, 8]. High-volume-disease patients with metastatic PCa have a poor prognosis, but we often see patients who live longer than what is reported in the literature. Stratification of metastatic PCa patients to identify high-risk PCa patients who should receive aggressive treatment is needed.

Serum alkaline phosphate (ALP) is a significant prognostic factor in metastatic PCa. ALP flare was first reported in 1996 [9] and may be useful for early diagnosis of patients in whom the disease is likely to progress rapidly and who would potentially benefit from aggressive treatment. This study focused on patients with *de novo* bone metastatic PCa who had progressed to CRPC and determined if the ALP flare ratio (ALP at 1 month after ADT/initial ALP value) predicted their OS. If metastatic disease patients with highly malignant disease could be identified, more aggressive treatment could be provided to the patients most likely to benefit.

## 2. Materials and Methods

One hundred and seventy-two patients of metastatic PCa had arrived at the Teikyo University Chiba Medical Center between 2008 and 2017, and 162 patients had been diagnosed with bone metastasis. We could have retrospectively assessed the ALP levels of 119 patients before and after 1 and 3 months of starting ADT. Clinical staging was evaluated according to the 2017 TNM classification. We used PCWG2/3 criteria to define CRPC. All patients had undergone transrectal needle biopsy to diagnose PCa pathologically, and the Gleason score was determined. All patients were treated with ADT including surgical castration and a luteinizing-hormone-releasing hormone agonist or antagonist with or without antiandrogen bicalutamide (80 mg daily). Fifty-two patients who had progressed to CRPC had been treated with next-generation ART (abiraterone acetate or enzalutamide) or docetaxel (Table 1). Written consent for PCa biomarker detection was obtained from each patient (Teikyo University Review Board No. 14-016).

The serum levels of PSA, ALP (normal range 87-304 U/L), hemoglobin, and lactate dehydrogenase (LDH) at diagnosis; ALP at 1 and 3 months after starting ADT; and PSA nadir were measured. A flare of ALP was defined as any increase in serum ALP above baseline at 1 month after ADT. Time to CRPC was defined as the time from the start of ADT to clinical or PSA progression (≥2.0 ng/mL). OS was defined as the time from the start of ADT until death from PCa or any cause.

Univariate comparisons between ART- and docetaxel-treated patients were assessed using chi-squared tests or Wilcoxon tests. Time to CRPC and OS were analyzed using the Kaplan–Meier method and the log-rank test. Univariate and multivariate Cox regression analyses of the available prognostic variables (age, Gleason score, PSA, PSA nadir, baseline ALP, value of ALP flare at 1 month after ADT, LDH, hemoglobin, and testosterone levels) were conducted to identify the significant prognostic factors. Statistical analyses were performed using JMP software version 11 for Windows (SAS Institute, Inc., Cary, NC). All tests were two sided, with significance accepted at *p* < 0.05.

## 3. Results

**Figure 1:**
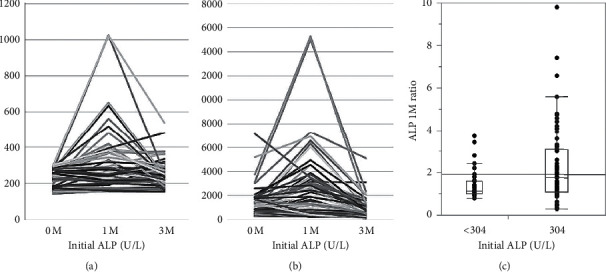
ALP change at 1 and 3 months after starting ADT in patients with (a) initial ALP levels less than 304 U/L and (b) initial ALP levels more than 304 U/L. (c) The ALP flare ratio was the ALP value 1 month after ADT divided by the ALP value at diagnosis. Groups were divided according to initial ALP levels within the normal range (<304 U/L) or higher. The median peak ALP flare ratios were 1.11 and 1.74 in patients with an initial ALP less than 304 U/L and more than 304 U/L, respectively (*p* < 0.0001).

The change in ALP levels after initiation of ADT was retrospectively investigated in 119 HSPC patients with bone metastases. Most patients showed a peak ALP flare at 1 month after ADT (Figure 1). Even 83.3% of patients within the normal ALP range (87–304 U/L) showed a peak 1 month after ADT (Figure 1(a)), as did 85.9% of patients with an initial ALP more than 304 U/L before ADT (Figure 1(b)). The median peak ALP flare ratios (ALP 1 month after ADT/initial ALP level) were 1.33 in all patients and 1.11 and 1.74 in patients with an initial ALP less than 304 U/L and more than 304 U/L, respectively (*p* < 0.0001) (Figure 1(c)). In 119 patients, ALP levels had decreased at 3 months in 88 patients (73.9%), equal to peak ALP in 16 patients (13.4%), and still increased at 3 months after ADT in 15 patients (12.6%).

Of the 119 metastatic HSPC cases, 95 patients developed to CRPC. Fifty-two patients were treated with ART (*n* = 35) or docetaxel (*n* = 17). Seventeen patients were treated with first-generation antiandrogen flutamide. The remaining 26 patients could not have life-prolonging treatment because of their advanced age, fragility, and poor performance status. Four in 35 patients treated with ART had sequential docetaxel treatment, and 10 in 17 patients treated with docetaxel sequentially were treated with ART.

The patients' characteristics are listed in Table 1. The median follow-up periods were 45.3 and 34.0 months in the ART and docetaxel groups, respectively. Baseline characteristics such as the Gleason score, *T* stage, presence of visceral metastasis, pretreatment PSA, LDH, ALP, hemoglobin, calcium, testosterone, and PSA nadir were similar between ART- and docetaxel-treated patients. The age at diagnosis in the ART group was greater than in the docetaxel group (75 vs. 67 years of age, respectively, *p*=0.0149).

**Figure 2:**
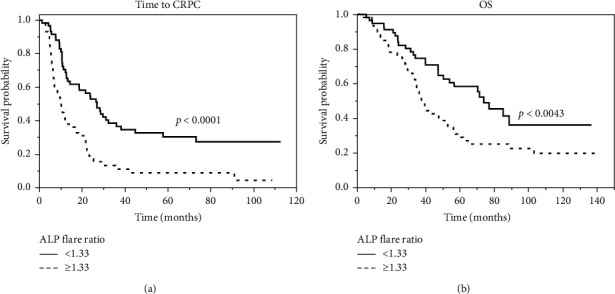
Kaplan–Meier curves showing time to castration-resistant prostate cancer (CRPC) (a) and overall survival (OS) (b) according to an ALP flare ratio less than 1.33 or 1.33 or more.

The median OS of the patients with metastatic PCa was 52.3 months. The median times to CRPC from diagnosis were 14.2 and 10.1 months for those with an ALP flare ratio less than 1.33 and 1.33 or more, respectively (*p* < 0.0001) (Figure 2(a)). The median OS from diagnosis was 73.8 and 37.6 months in those with an ALP flare ratio less than 1.33 and 1.33 or more, respectively (*p* < 0.0043) (Figure 2(b)).

**Figure 3:**
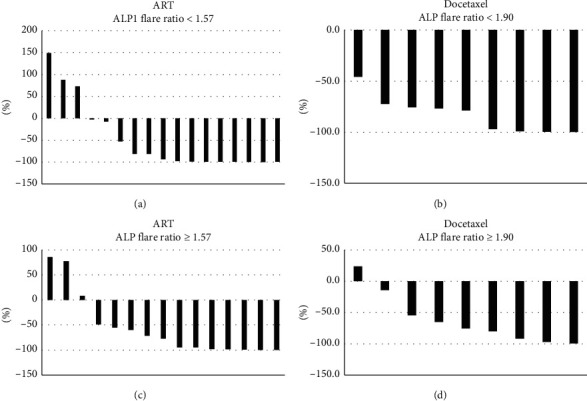
Waterfall plot of the best PSA decline after CRPC treatment compared with an ALP flare ratio less than 1.57 and 1.57 or more in the group that received second-generation androgen receptor targeting therapy (ART) (a, b) and an ALP flare ratio less than 1.90 and 1.90 or more in the group that received docetaxel (c, d).

In this study, we focused on the role of the ALP flare ratio in predicting the response to CRPC treatment (ART and docetaxel). The median ALP flare ratios were 1.57 and 1.90 in ART- and docetaxel-treated patients as the first-line CRPC treatment, respectively. PSA declined at least 50% with ART treatment as first-line therapy for CRPC in 70.5% and 80% of patients with an ALP flare ratio of less than 1.57 and 1.57 or more, respectively (*p*=0.831) (Figures 3(a) and 3(b)). PSA declined at least 50% with docetaxel treatment as the first-line therapy for CRPC in 90% and 77% of patients with an ALP flare ratio of less than 1.90 and 1.90 or more, respectively (*p*=0.428) (Figures 3(c) and 3(d)). These data indicate that the PSA decline was not associated with the ALP flare ratio.

**Figure 4:**
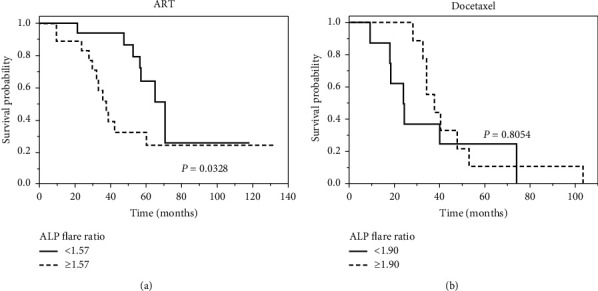
Kaplan–Meier curves showing overall survival (OS) after starting ADT with androgen receptor targeting therapy (ART) (a) or docetaxel (b) as the first-line CRPC treatment, comparing an ALP flare ratio at 1 month less than 1.57 and 1.57 or more (a) and less than 1.90 and 1.90 or more (b).

In patients treated with ART, the median OS from diagnosis was 70.3 and 37.3 months in patients with an ALP flare ratio less than 1.57 and 1.57 or more, respectively (*p*=0.0328) (Figure 4(a)). On the other hand, in patients treated with docetaxel, the median OS from diagnosis was 31.9 and 34 months in patients with an ALP flare ratio less than 1.90 and 1.90 or more, respectively (Figure 4(b)), a difference that was not significant. Progression-free survival (PFS) after ART and docetaxel was not statistically different between ALP flare levels high and low in both ART and docetaxel groups (Supplementary Figure 1).

Univariate analysis showed that the Gleason score, PSA nadir, ALP at diagnosis, ALP flare ratio 1 month after ADT, and LDH were significant predictors of OS (Table 2). The PSA nadir, ALP flare ratio, and LDH showed significant higher-risk mortality both in univariate and multivariate analyses. Specifically, the ALP flare ratio was the most significant factor to predict OS in patients with metastatic PCa (multivariable hazard ratio 1.82, 95% confidence interval 1.350–2.512, *p*=0.0001) (Table 2).

### 3.1. Comments

Performance status, pain intensity, hemoglobin, PSA, Gleason score, ALP, LDH, and the presence of visceral metastases are factors that predict the prognosis of metastatic HSPC or CRPC [10–12]. ALP is a bone formation marker, and higher levels of ALP above the normal range predict the incidence of skeletal complications or the prognosis of metastatic PCa patients [13–17]. ALP is also a significant prognostic factor to predict survival after docetaxel treatment [11] and new hormonal therapy including abiraterone [18, 19]. ALP flare was first reported in malignancy with osteoblastic bone metastasis [20, 21]. In our data, the peak of the ALP flare was 1 month after initiation of ADT, similar to previous reports. The significance of an ALP flare for predicting OS in PCa was first reported in 1996; elevation of ALP ≥ 50% above baseline after initiation of ADT is a useful predictor to identify patients who would progress rapidly after orchiectomy and who would potentially benefit from aggressive treatment [9]. Robinson et al. reported that baseline ALP, ALP flare, and ALP at 6 months were significantly associated with death from PCa within 3 years [15]. In our data, OS was better in patients with an ALP flare ratio less than 1.33 compared a ratio of 1.33 or higher. The PSA response following ART and docetaxel as CRPC treatment was not significantly different between a lower or higher ALP ratio. No data have been reported about ALP flare and response to next-generation treatment. The OS was poor in patients with an ALP flare ratio of 1.57 or more compared to those with a ratio less than 1.57, suggesting that the ALP flare ratio might be a prognostic marker in ART-treated patients. We also found that, in patients with docetaxel treatment for CRPC, the OS was not significantly different according to the ALP flare ratio. These data suggest that docetaxel may overcome the aggressiveness of the disease.

An important conclusion is that only 1 month after induction of ADT, we can predict who will progress early and have a poor prognosis. The present data confirmed that a higher ALP flare ratio (≥1.57) predicts worse OS in patients with metastatic PCa. The ALP flare ratio was the most significant marker to predict the prognosis of patients with metastatic PCa (Table 2).

The mechanisms of ALP flare have not been fully elucidated, but activation of osteoblasts may be initiated by any cancer pathway by treatment [13, 20, 21]. An ALP flare could be a reliable marker to select patients who may benefit from aggressive treatment for metastatic PCa.

This study has several limitations. First, a small number of patients were analyzed because this study was conducted in a single academic center and included patients in the era of docetaxel, which is after 2008 in Japan. Second, the treatment timing and selection of treatment for CRPC (ART or docetaxel) depend on each doctor's decision without a precise protocol and may have resulted in a bias in patient selection, especially for initiation of docetaxel. Selection of treatment depends on the performance status, comorbidities, and the age of the patients and was based on real-world clinical practice rather than clinical trials. Third, due to its retrospective design, the data might not be extrapolated to the clinical practice because it is not validated. The results of this study would be better if validated by a large-scale clinical trial with balanced randomization.

## 4. Conclusions

This study indicated that the ALP flare ratio after the first-line ADT might be a possible prognostic factor, being an early and easily collectable variable at diagnosis of PCa patients with bone metastasis. Thus, precise measurements of ALP after initiation of ADT may identify patients with a poor prognosis at an earlier time before the patients have progressed to CRPC and allow selection of patients for whom it would be better to start chemotherapy first other than ART.

## Figures and Tables

**Table 1:** Baseline characteristics of patients treated with ART and docetaxel.

Variables	ART	Docetaxel	*p*
Number of patients	35	17	
Age (years)	75 (60–92)	67 (56–89)	0.0149
Clinical stage T2	2	0	0.46
T3	14	6	
T4	19	11	
Visceral metastasis	5	1	0.63
Gleason score ≤8	14	9	0.44
Gleason score ≥9	21	8	
PSA (ng/mL)	472 ± 497	277 ± 701	0.98
LDH (U/L)	218 ± 22	187 ± 15	0.22
ALP (U/L)	513 ± 249	617 ± 154	0.65
Hemoglobin (g/dL)	13.6 ± 0.3	14.1 ± 0.5	0.79
Calcium (mg/dL)	9.1 ± 0.09	9.1 ± 0.14	0.63
Testosterone (ng/mL)	3.5 ± 0.25	3.8 ± 0.38	0.55
PSA nadir (ng/mL)	0.66 ± 3.11	1.36 ± 4.33	0.95
Follow-up duration (months)	45.3 ± 4.7	34.0 ± 5.5	0.21

ART: androgen receptor targeted therapy, PSA: prostate-specific antigen, LDH: lactate dehydrogenase, ALP: alkaline phosphatase.

**Table 2:** Uni- and multivariate analysis of impacts of various parameters on overall survival from diagnosis in patients with metastatic prostate cancer.

Variables	Univariate			Multivariate		
	HR	95% CI	*p*	HR	95% CI	*p*
Age	1.009	0.975–1.043	0.99	1.02	0.970–1.073	0.4208
Gleason score	1.804	1.273–2.560	0.0009	1.34	0.681–2.611	0.3918
PSA at diagnosis	1	0.999–1.000	0.973	0.999	0.9991–0.9996	0.0247
PSA nadir	1.002	1.000–1.003	0.0228	1.022	1.003–1.042	0.0227
ALP at diagnosis	1.0002	1.0000–1.0004	0.007	0.999	0.9994–1.0001	0.4046
ALP flare ratio	1.151	1.012–1.007	0.0337	1.885	1.374–2.647	<0.0001
LDH at diagnosis	1.005	1.002–1.007	0.0003	1.004	1.0004–1.0088	0.0311
Hemoglobin	0.911	0.812–1.033	0.1441	0.859	0.6943–1.0781	0.1847
Testosterone	0.932	0.724–1.199	0.587	0.8251	0.6033–1.1309	0.229

ART: androgen receptor targeted therapy, PSA: prostate-specific antigen, LDH: lactate dehydrogenase, ALP: alkaline phosphatase.

## Data Availability

No data were used to support this study.

## Supplementary Materials

Supplementary Materials Supplementally Figure 1: Kaplan–Meier curves showing progression-free survival (PFS) after starting ADT with androgen receptor targeting therapy (ART) (A) or docetaxel (B) as the first-line CRPC treatment, comparing an ALP flare ratio at 1 month less than 1.57 and 1.57 or more (A) and less than 1.90 and 1.90 or more (B).
