# Supplementary figures and images for: High Serum Alkaline Phosphatase Flare after First-Line Androgen Deprivation Therapy Predicts Poor Prognosis in Metastatic Prostate Cancer Patients Treated with Second-Generation Androgen Receptor Targeted Therapy
Source: Prostate Cancer. 2021 Apr 8;2021:5574067. doi: 10.1155/2021/5574067 (PMC8052161; doi:10.1155/2021/5574067)

Supplemental Figure 1


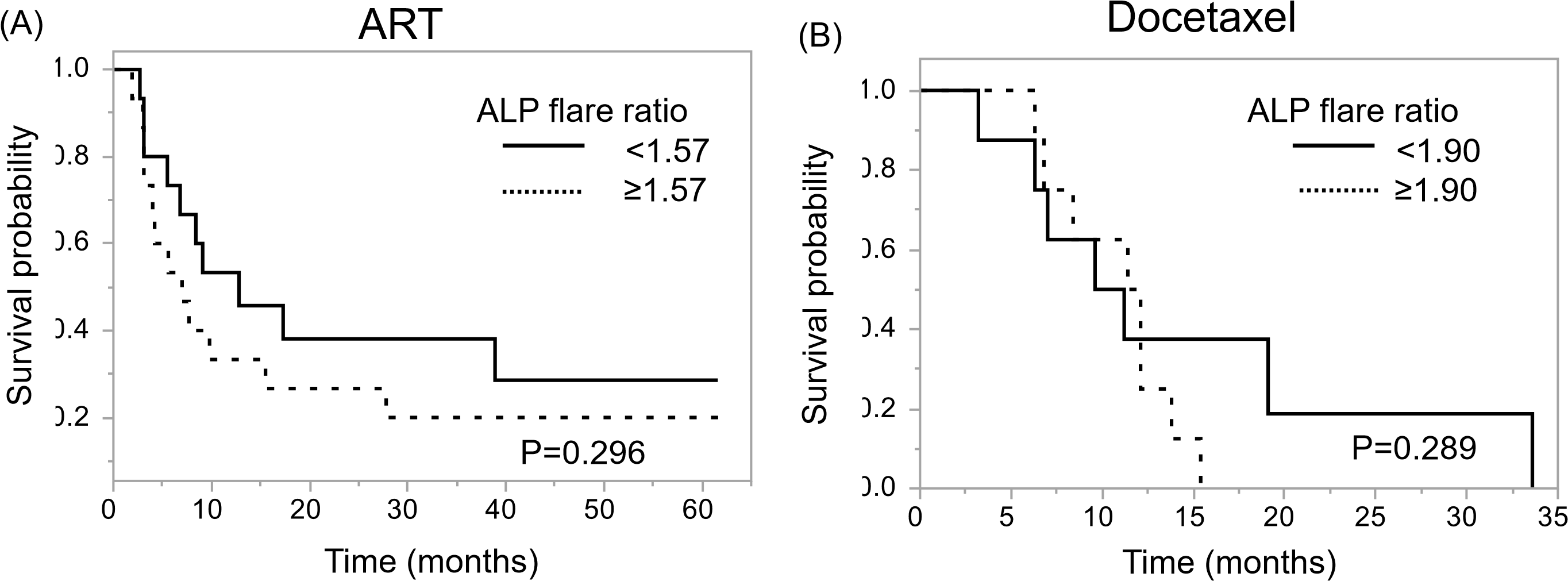

Supplement: Supplementary Materials — Supplementally Figure 1: Kaplan–Meier curves showing progression-free survival (PFS) after starting ADT with androgen receptor targeting therapy (ART) (A) or docetaxel (B) as the first-line CRPC treatment, comparing an ALP flare ratio at 1 month less than 1.57 and 1.57 or more (A) and less than 1.90 and 1.90 or more (B). [file 5574067.f1.docx]
